# Vaccination and Re-Vaccination

**Published:** 1865-03

**Authors:** 


### VACCINATION AND RE-VACCINATION.

At the last meeting of the Buffalo Medical Association remarks were made by different members upon the importance and necessity of vaccination and re-vaccination; the Association regarding the prevalence of small pox as unnecessary and disgraceful to the medical police of cities. Since this disease is prevailing in large cities throughout the country, and in some degree in Buffalo, though to a much more limited extent than in most other places, a committee was appointed to present the views of the profession upon this subject to the board of health, common council and citizens, with the view to obtain action in a matter which they regard as vital to the health and safety of the public.

The committee in presenting the facts which are sustained by the most careful and extensive observation would quote the following conclusions, which have been made the basis of municipal regulations in other cities, and are embodied in a Report by a Committee of the American Medical Association, held in New York June, 1864, and also presented to the State Medical Society in a Prize Essay by A. N. Bell, M. D., of Brooklyn:

"1.—That vaccination is immensely protective against epidemic diseases generally, and against small-pox in particular; and against death by small-pox, the protective power of vaccination is almost perfect.

2.—That of any number of persons who have had unmodified small-pox, the proportion wholly protected from a second attack at adult age, is 43 per cent., while 57 per cent. are liable to it again in some form or other.

3.—That out of any number of adult persons who have good marks of vaccination, $40\frac{1}{2}$ per cent. are perfectly protected; while $59\frac{1}{2}$ per cent. are susceptible to varioloid, or to re-vaccination to such a degree as to render their protection perfectly complete.

4.—That the degree of protection afforded by previous unmodified small-pox, from a second attack, is only $2\frac{1}{2}$ per cent. greater than the protection afforded by vaccination; a proportion too small to be regarded as any evidence of real difference in protective power, and reasonably attributable to spurious or impaired vaccination from a variety of causes, such as vaccination during the progress of other diseases, injury of the vesicle or defective lymph.

5.—That out of any number of adult persons with imperfect marks of vaccination, 23 per cent. only are protected, while 77 per cent. are liable to small-pox or varioloid.

6.—The liability to varioloid after ten years of age, of persons vaccinated under three years of age; and the increased liability again from fifteen to twenty-five years of age, of persons vaccinated or re-vaccinated at from ten to fifteen years of age, demonstrates that, generally, protection by vaccination under twenty-five years of age is complete for about seven years only. Subsequent to twenty-five years of age, protection is complete for a greater length of time, proportionate to the age of the individual at the time of the vaccination.

7.—That during the prevalence of an epidemic of small-pox there is increased susceptibility to the disease, and the degree of protection from previous vaccination is proportionately lessened. Under such circumstances, therefore, it is incumbent to re-vaccinate at intervals not exceeding five years; and in cases of certain exposure as soon as practicable thereafter, as there is abundant evidence of the protective power of vaccination and re-vaccination even as late as the fourth day after exposure to small-pox.

8.—Protection is known to be complete only when fresh vaccine lymph from a perfect vesicle fails to take on re-vaccination."

The Committee of the Buffalo Medical Association in view of the above well sustained conclusions, would respectfully urge upon the Board of Health and Common Council, that immediate provision be made, by the employment of competent and faithful men, for the vaccination and re-vaccination of all who may be found unprotected from small-pox, and are poor, negligent, inattentive, or ignorant of the efficacy and value of vaccination. They would also call the attention of the public generally to the importance of vaccination and re-vaccination until they are sure that fresh vaccine lymph from a perfect vesicle will not take on re-vaccination. When this evidence of vaccination has been obtained the protection is perfect; it cannot be known to be complete without it.

JULIUS F. MINER,<br>THOMAS F. ROCHESTER, } *Committee.* { PHINEAS H. STRONG,<br>SANDFORD EASTMAN,

